# Transforming the Future of Digital Health Education: Redesign of a Graduate Program Using Competency Mapping

**DOI:** 10.2196/54112

**Published:** 2024-10-31

**Authors:** Michelle Mun, Sonia Chanchlani, Kayley Lyons, Kathleen Gray

**Affiliations:** 1 Centre for Digital Transformation of Health, Faculty of Medicine, Dentistry and Health Sciences, The University of Melbourne, 700 Swanston Street, Parkville, Melbourne, 3010, Australia, 61 9035 5553; 2 Melbourne Dental School, Faculty of Medicine, Dentistry and Health Sciences, The University of Melbourne, Melbourne, Australia; 3 Melbourne Medical School, Faculty of Medicine, Dentistry and Health Sciences, The University of Melbourne, Melbourne, Australia

**Keywords:** digital health, digital transformation, health care, clinical informatics, competencies, graduate education

## Abstract

Digital transformation has disrupted many industries but is yet to revolutionize health care. Educational programs must be aligned with the reality that goes beyond developing individuals in their own professions, professionals wishing to make an impact in digital health will need a multidisciplinary understanding of how business models, organizational processes, stakeholder relationships, and workforce dynamics across the health care ecosystem may be disrupted by digital health technology. This paper describes the redesign of an existing postgraduate program, ensuring that core digital health content is relevant, pedagogically sound, and evidence-based, and that the program provides learning and practical application of concepts of the digital transformation of health. Existing subjects were mapped to the American Medical Informatics Association Clinical Informatics Core Competencies, followed by consultation with leadership to further identify gaps or opportunities to revise the course structure. New additions of core and elective subjects were proposed to align with the competencies. Suitable electives were chosen based on stakeholder feedback and a review of subjects in fields relevant to digital transformation of health. The program was revised with a new title, course overview, course intended learning outcomes, reorganizing of core subjects, and approval of new electives, adding to a suite of professional development offerings and forming a structured pathway to further qualification. Programs in digital health must move beyond purely informatics-based competencies toward enabling transformational change. Postgraduate program development in this field is possible within a short time frame with the use of established competency frameworks and expert and student consultation.

## Introduction

In contrast to simple digitization of processes, digital transformation describes the “comprehensive reorientation of an industry, including its business models, due to the coming of age of digital technologies” [1]. In health care, digital technologies have attracted considerable investment for their potential to reduce costs, improve patient experience, and clinician and system efficiency [2]. However, potential digital health interventions can experience “pilotitis” as innovators and health systems can lack the reciprocal clarity of roles and processes to be able to successfully design, launch, and scale a robust product [3]. This fragmentation explains the observation that while diverse innovative digital health interventions have proliferated in the last 50 years [4], the hope for transformational change and increased value-add of health systems has not yet been delivered [5].

Alongside global recognition of the importance of digital health [6], the domains of digital health and health informatics have become areas of increasing focus for education and workforce development. In Australia, the newly published National Digital Health Capability Action Plan (CAP) and institutional education strategies have a significant role to play in building digital health capability across the health workforce [7-9]. Across a 7-year roadmap, the CAP has outlined priority actions including the development of specialist digital health career pathways, specialist digital health courses, and continuing professional development opportunities for clinicians, informaticians, service management, and related roles in the health sector.

However, the digital transformation of health care cannot be driven by 1 sector alone. For this reason, innovation centers have been established globally in recent years to facilitate collaboration between all stakeholders involved in digital health [3]. In Australia, the University of Melbourne runs multidisciplinary digital health programs through The Center for Digital Transformation of Health, established in 2019 with the vision of “connecting digital innovation to health” [10]. The center sits within the Faculty of Medicine, Dentistry and Health Sciences and operates in conjunction with the School of Computing and Information Systems, Faculty of Engineering and IT. In 2023, the authors of this paper were commissioned for 3 months to redesign and adapt the existing Graduate Certificate in Health Informatics and Digital Health to meet contemporary national and international digital health standards and align with key center vision and mission objectives.

This viewpoint describes the realignment of the existing Graduate Certificate in Health Informatics and Digital Health with the philosophy of “digital transformation,” building on the internationally recognized American Medical Informatics Association (AMIA) clinical informatics competency framework [11,12]. The methodology may be used as a blueprint to aid the development of future digital health programs.

## Methods

### Market Context

**Figure 1.**
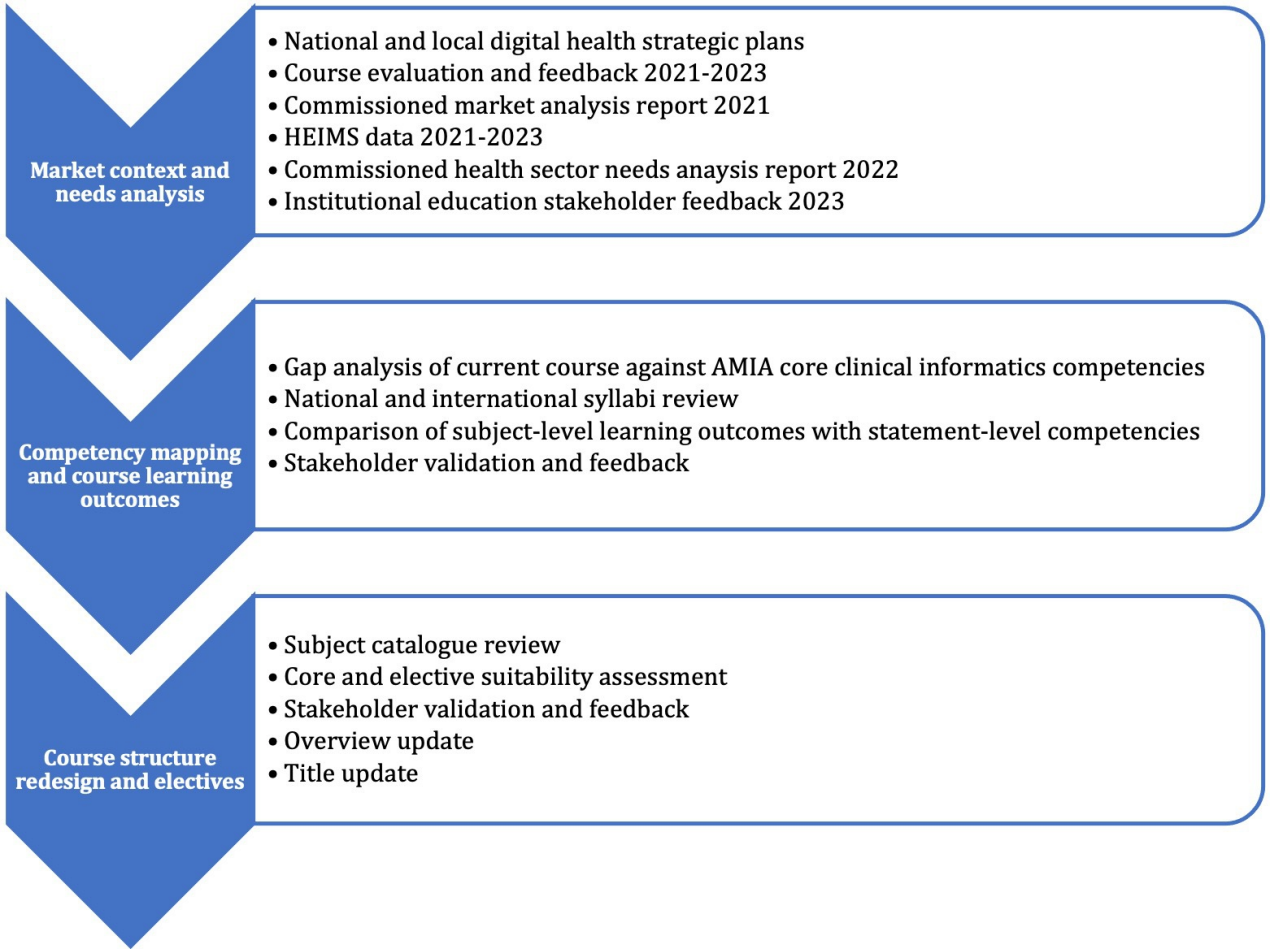
Process for evaluation and redesign of the digital health postgraduate program. AMIA: American Medical Informatics Association; HEIMS: Higher Education Information Management System.

The market needs analyses and student feedback informed subsequent stages of competency mapping and course structure review (Figure 1).

A market scan was conducted using data from the Higher Education Information Management System, from the University of Melbourne Department of Education and Training. Over the past 5 years, universities across Australia have seen increased interest in students pursuing higher professional certification in digital health. Many new postgraduate offerings in digital health, eHealth, health information management, and health and clinical informatics have been created, with 60‐70 new enrollments in graduate certificates offered in Queensland and Victoria in 2023 alone [13].

Several commissioned needs assessments, market analysis reports, and student reflections within course evaluations were undertaken by the center between 2021 and 2023 to assess future directions for offering digital health professional development, degree, or award programs. The analyses confirmed the challenges of the changing clinical landscape and increased enrollment trends in national graduate certificates in digital health, as well as the presence of an emerging market for people entering or transitioning into the field of digital health. In October 2020, some students (n=14, 20% response rate) enrolled in the University of Melbourne Master of Information Systems Health specialization and the graduate certificate indicated that they were looking for new digital health work opportunities (10 of 14, 71.4% response rate). The survey respondents recommended stronger education in key entry-to-practice degrees and incentives for continued professional development programs.

A recurring theme indicated current clinical career pathways in digital health are not dependent on formal professional certification. Of the 699 advertised digital health–related jobs found over a 3-month period, between October 12, 2020, and January 18, 2021, of which 130 positions were advertised in the state of Victoria, there was significant variation in the range of qualifications, as well as the specialized knowledge and skills relevant to digital health. Short course participant data also highlighted that clinicians are more inclined to consider a graduate certificate if it helped progress their career, with top subjects of interest in domains of data science and artificial intelligence (“machine learning, artificial intelligence and big data” and “data analytics, data linkage, power BI and R”), and the development, implementation, or evaluation of digital health interventions.

Key quotes from participants included “I think you need some ability to put skills learnt into practice in a guided way to really make an impact” and “Theory is just more theory and many of us are way beyond that. Those wanting to do this course would be wanting to make a change, not theorise about it.”

In summary, and with consideration to the accelerating pace of digital health technology development, it was anticipated that the observations heralded ongoing and increasing interest in this field.

### Competency Mapping

The University of Melbourne’s Graduate Certificate in Health Informatics and Digital Health, offered at the postgraduate level, or Australia Qualifications Framework level 8, sits above the professional certificate, and upon completion can be streamlined into a relevant master’s degree. The existing certificate consisted of 3 core subjects in biostatistics, health informatics methods, and critical thinking with analytics, alongside limited elective subject options.

To ensure the certificate had core alignment with international informatics standards, SC and MM examined the learning outcomes in subjects in the existing certificate and compared them to the AMIA Core Clinical Informatics Competencies. The Australian Health Informatics Competency Framework (AHICF), developed by the national peak body for informatics, the Australasian Institute of Digital Health (AIDH) [14] was also examined, and syllabi of comparable national and international graduate certificates, sourced from institutional websites, were considered for completeness. The initial mapping phase consisted of a direct comparison of subject-level learning outcomes with statement-level competencies in the AMIA framework. Subsequently, the results were summarized and presented to an expert panel of subject coordinators and center leadership to confirm the accuracy of the mapping and identify further gaps and opportunities.

Results from the competency mapping and the panel interview were used to inform the decision about whether there were existing University of Melbourne subjects that could be used to complement missing competencies, or if there was an opportunity to develop a new subject in digital health, or both. Subjects in the certificate should also be included if they could be accredited toward a relevant master’s degree, should the certificate student choose to continue with a higher qualification.

### Course Learning Outcomes and Course Structure Redesign

Course-level learning outcomes were developed with consideration to (1) core clinical informatics competencies and (2) desired skills and knowledge beyond clinical informatics, that could equip a graduate to navigate the digital transformation of health care. The latter was derived from course participant feedback, as outlined, as well as key concepts from digital transformation literature:

- “A multi-stakeholders perspective (which) is critical to understanding properly how, in practice, the various players of a (healthcare) ecosystem (patients, pharmaceutical companies, hospitals, public agencies, and many more) exploit (digital transformation) technologies and means to quality of care, value creation, and many more managerial issues” [15]
- Reducing roadblocks that may slow innovation in health systems, including “aligning cross-departmental stakeholders (information technology, security, risk, legal, etc)” [3]
- Introducing the concept of learning health systems, in which “science, informatics, incentives, and culture are aligned for continuous improvement and innovation, with best practices seamlessly embedded in the care process, patients and families as active participants in all elements, and new knowledge is captured as an integral by-product of the care experience [16].”

Core subjects were selected from the results of the competency mapping, and their subject-level learning outcomes were reviewed to ensure accordance with the course-level learning outcomes.

### Electives for Digital Transformation of Health

The University Handbook, an online catalog of courses and programs, was searched for subjects that could be suitable for inclusion in the certificate as elective subjects. Inclusion criteria included subjects in domains relevant to data science and statistics, product development, business, leadership and change management, consumer participation in health care, research methods, sustainability, or ethics or legal subjects. A total of 79 subjects were identified. Upon closer consideration, 50 were excluded for the reasons of limited relevance to digital health; required pre-requisite subjects that could not be taken within the constraints of the graduate certificate; discontinued subject; or timing or delivery of the subject not suitable (eg, not semester-long or on-campus only, whereas the certificate required hybrid or online subjects).

As it was intended that the graduate certificate could be used to build a career pathway in digital health, the remaining subjects were assessed for their suitability to scaffold toward a master’s pathway. Coordinators of eligible subjects were contacted to determine their availability and interest to be part of the new graduate certificate. After approvals were received, subjects were chosen for their eligibility to form a pathway from professional certificate, graduate certificate, to master’s pathways in public health, information systems, and clinical Research.

## Results

The mapping and interview phases revealed that many core informatics competencies were already covered within the existing certificate, but there were opportunities to include advanced content about data science, machine learning, and artificial intelligence; the development, implementation, and evaluation of digital health interventions, digital transformation of health care systems, and indigenous data governance. These topics have become increasing areas of interest and debate in recent years, and their need for inclusion in the certificate was evident if the certificate was to both align with recognized standards and be relevant to the modern digital health landscape. Additionally, the mapping process highlighted the need for a more structured approach to the certificate design, which the center was in a position to provide given the depth and diversity of expertise available.

The new certificate consists of 2 core subjects that were previously electives, that had been identified as achieving the most comprehensive range of AMIA Core Clinical Informatics Competencies (Multimedia Appendix 1). Further mapping to AHICF competencies confirmed alignment with national clinical informatics competencies (Multimedia Appendix 2). The Applied Learning Health Systems subject additionally had a strong focus on practical multidisciplinary learning for the digital transformation of health. The certificate title, course overview, and course-level learning outcomes were updated to align with the skills and competency requirements of the changing market (Textbox 1).

The proposed course-level learning outcomes could now map upstream to the 5 AMIA Clinical Informatics domains (Textbox 2, Table 1) and downstream to subject-level intended learning outcomes of the 2 core subjects (Table 2). Considering the close alignment of the AMIA and AHICF frameworks, for simplicity, the table shows mapping to AMIA competencies only.

A total of 13 electives were identified that would complement the core subjects and allow participants the flexibility to build knowledge and skills in 1 of the self-identified areas of data science and statistics, product development, business, leadership and change management, consumer participation in health care, research methods, sustainability, or ethico-legal contexts in digital health (Table 3). Students would be able to choose 2 elective subjects to complement the core subjects.

To reflect the center’s vision of translating digital health innovations into clinical practice, the final metamorphosis of the course included a strategic title change from Graduate Certificate of Health Informatics and Digital Health to Graduate Certificate in Digital Transformation of Health.

Textbox 1. **Comparison of previous and newly developed course-intended learning outcomes.**

**Previous course-level intended learning outcomes**

On completion of this course, graduates will be able to:

- Communicate knowledgeably about core health and biomedical informatics concepts, tools and methods, and methods.
- Critically evaluate approaches to information systems and information technology in contemporary health care in Australia and internationally.
- Develop an integrated understanding of how digital data, information, and knowledge are generated and managed for clinical care, biomedical research, public health, and health policy and planning.

**New course-level intended learning outcomes**

On completion of this course, graduates will be able to:

- Describe how contemporary digital health technologies can be integrated into health care practice in terms of their effect on safety and quality, access and equity, continuity of care, effectiveness, and consumer empowerment.
- Critically evaluate the generation, governance and use of digital data, information and knowledge, including legal and ethical considerations, in the context of electronic health records, clinical decision support systems, virtual care, mobile health, and machine learning and artificial intelligence applications in health.
- Apply the concept of a learning health system and processes of problem assessment, data analysis, design thinking, implementation science, and evaluation frameworks to digital health initiatives in specific contexts.
- Apply principles of governance, leadership, change management, and strategic planning to integrate digital health initiatives and innovation within organizations, across communities and health care systems.

Textbox 2. **The American Medical Informatics Association clinical informatics competency domains.**

- Fundamentals
- Improving care delivery and outcomes
- Enterprise information systems
- Data governance and data analytics
- Leadership and professionalism

**Table 1.** Relationship of clinical informatics competencies and new course-level intended learning outcomes.

Course-level intended learning outcomes (CILO)	Relevance to AMIA^a^ domains
CILO1: Describe how contemporary digital health technologies can be integrated into health care practice in terms of their effect on safety and quality, access and equity, continuity of care, effectiveness, and consumer empowerment.	AMIA1, AMIA2, and AMIA3
CILO2: Critically evaluate the generation, governance, and use of digital data, information, and knowledge, including legal and ethical considerations, in the context of electronic health records, clinical decision support systems, virtual care, mobile health, and machine learning and artificial intelligence applications in health.	AMIA2, AMIA3, and AMIA4
CILO3: Apply the concept of a learning health system and processes of problem assessment, data analysis, design thinking, implementation science, and evaluation frameworks to digital health initiatives in specific contexts.	AMIA1 and AMIA2
CILO4: Apply the principles of governance, leadership, change management, and strategic planning to integrate digital health initiatives and innovation within organizations, across communities, and health care systems.	AMIA5

a AMIA: American Medical Informatics Association.

**Table 2.** Relationship of new course-level and subject-level intended learning outcomes.

Core subject title and subject-level intended learning outcomes (SILO)		Relevance to course-level intended learning outcomes (CILO)
**Digital transformation of health**		
	SILO1: Explain complex aspects of the structure of health care, including the roles of patients, various professionals, insurance companies and governments.	CILO1
	SILO2: Describe the implications of the generation and the use of biomedical data, information, and knowledge within a variety of relevant systems and settings.	CILO2
	SILO3: Demonstrate the understanding of how core digital health technologies work, through practical activities with simulations of tools such as electronic health records, clinical decision support systems, patient portals, and mobile apps and wearable sensors.	CILO1 and CILO2
	SILO4: Critically analyze how various digital technologies can optimize information use within health care and summarize the potential risks associated with these solutions.	CILO1
	SILO5: Apply ethical frameworks and conceptual models to critique contemporary digital health practices and trends.	CILO1
**Applied learning health systems**		
	SILO1: Appraise emerging trends and approaches in digital health and informatics.	CILO1 and CILO2
	SILO2: Illustrate how concepts of the learning health system can be applied to your current workplace and role.	CILO3
	SILO3: Outline potential activities in a learning health system project starting with data access and analysis—through designing a virtual care model—and ending with evaluation, implementation, and transformation.	CILO1, CILO3, and CILO4
	SILO4: Create a proposal for a learning health systems (LHS) project that could be implemented at your current or future workplace, which applies digitally enabled LHS concepts.	CILO3 and CILO4

**Table 3.** Elective subjects for digital transformation of health.

Elective title	Elective overview
Digital health informatics methods	Overview of major health informatics research areas and methods that contribute to quality improvement, scientific research, and technological innovation in health care and biomedicine.
Biostatistics	Introduction to the fundamental concepts of statistics and the essential methods required to equip students to perform basic statistical analyses and interpret research findings in the public health setting.
Digital health for consumers	Explores wise use of consumer health technologies through dimensions of consumer digital health literacy, global consumer health technology marketplace, lived experiences of active users, and scenarios where consumers are partners in designing and using digitally enabled learning health systems.
Leading health care change for impact	Examines strategies for leading change in clinical settings and health care organizations.
Technology and aging	Examines ways in which recent technological advancements can revolutionize the experience, management, and future of aging.
Health care environment evaluation	Explores the complex, dynamic, interdisciplinary, and multipurpose nature of health care environments focusing on key dimensions of physical workspaces design, virtual work-spaces, and leadership and management practices.
Introduction to programming	Introduction to the fundamental concepts of computer programming and how to solve simple problems using high-level procedural language, with a specific emphasis on data manipulation, transformation, and visualization of data.
Law and emerging health technologies	Examine ways in which law is affecting, and being affected by, the latest advances in medical technology, including genetic, big data analytics, regenerative, therapeutic, artificial intelligence, and reproductive technologies.
Innovation and emerging technologies	Introduction to innovative and contemporary technology that has been recently developed and is currently used in clinical practice and research for the purposes of measurement, diagnosis, and prescription.
Sustainability and health care	Explores the need to urgently formulate adaptation and mitigation strategies, thereby addressing the global climate change emergency, through the lens of sustainable health care.
Natural language processing	Learn computational methods for working with text, in the form of natural language understanding and language generation to develop an understanding of the main algorithms used in natural language processing.
Machine learning applications for health	Introduction to different artificial intelligence applications in health, using different clinical data sources and computational techniques.
Indigenous data governance in health	Provides an overview of the scope of Indigenous data including governance, ethical health research, knowledge translation and evaluation, institutions, and data collections.

## Discussion

### Principal Results

The redesigned Graduate Certificate in Digital Transformation of Health occurs in the context of increasing awareness of the need to develop a digitally capable health workforce [7-9]. However, broader industry trends show that the digital health sector also contains diverse careers in data analysis, informatics, and application development, with early and mid-career professionals from clinical care, health management, and technology sectors keen for interactive, practical, and interdisciplinary learning [17]. Although the certificate was redesigned with clinical informatics competencies in mind, a high proportion of its students are likely to be from nonclinical backgrounds or nonphysician careers, based on the demographics of previous enrollments.

It was, therefore, the aim of the certificate to align with industry trends and provide flexibility for any professional interested in digital health transformation, not just clinicians, to tailor their learning. This was achieved by mapping the competency frameworks, which revealed 2 subjects that could be used as core subjects, allowing the certificate to be condensed from 3 to 2 cores and creating room for 2 elective subjects. The new “2 core plus 2 elective” structure allows the professional certificate to form a path to a graduate certificate and also allows a range of elective choices that are unique to the certificate. The newly identified electives were not only chosen based on participant feedback and framework gaps but also are aimed to empower health professionals to lead change within our complex adaptive health system. Newly identified electives with a focus on innovation and emerging technologies, indigenous data governance, sustainable health care, and machine learning applications will facilitate the next transformative phase within the industry.

The new title aligns with existing course offerings including the Professional Certificate of Digital Transformation of Health and organizational strategic vision. The program’s content also aligns with new industry trends strengthening management and leadership skills in the core Applied Learning Health Systems subject to allow for a spectrum of digital health pathways. This is critical as although many traditional degrees have set curricula and linear career pathways, learners in digital health come with vast differences in career backgrounds, qualification levels, expertise, amount of work experience, and intrinsic motivations for joining digital health. Consequently, they may end up applying their knowledge and skills to any part of the digital health ecosystem.

### Limitations

The multitude of certifications and career pathways in digital health reflects the demand from professionals to enter this pathway. However, the complexity of developing a program in this rapidly progressing field cannot be overstated. This viewpoint describes the practical needs assessment and redevelopment of a graduate program within the time constraints of an institutional schedule. Consultation for this project was informed by reports that included student evaluations but mainly occurred at the faculty educators and executive level. Given the diverse demographics of digital health learners, there is scope to continue co-design the program with past and potential future certificate students, as well as other major stakeholders in digital health such as consumer advocates.

Future work will focus on evaluating the acceptability and effectiveness of the structure and content of the certificate to these stakeholders. A robust evaluation process, modeled on the Kirkpatrick framework, is already in place within 1 core subject [18] and forms the model for evaluation for other subjects in the certificate. In addition, all University subjects undergo continuous evaluation using Student Learning Surveys, in accordance with University standards and processes [19]. As comprehensive program evaluation is critical for its long-term success, assessment of further approaches such as the creation of logic models [20] is underway.

Mapping the previous program to clinical informatics competencies and student feedback was efficient in reaching this point. Challenges ahead lie in maintaining the currency of the learning content and ongoing evaluation and improvement of the effectiveness of its curriculum, learning activities, and assessments. The program will continue to be reviewed against the progress of the CAP, the evolution of the digital health landscape in Australia, and insights from international colleagues and organizations. The next steps will include the development of a decision matrix to aid the prioritization and co-design of new subjects.

### Conclusions

A systematic refinement of this postgraduate program has been conducted to align with the center’s vision of digital innovation and transformation of health care. Through strategic alignment, competency mapping, and a pedagogical ethos, the transformed graduate certificate aspires to make a substantial impact on the evolving health care ecosystem.

## Supplementary material

10.2196/54112 Multimedia Appendix 1 Mapping of core subjects to American Medical Informatics Association competencies.

10.2196/54112 Multimedia Appendix 2 Mapping of core subjects to Australian Health Informatics Competency Framework competencies.

## Abbreviations

AHICF: Australian Health Informatics Competency Framework
AIDH: Australasian Institute of Digital Health
AMIA: American Medical Informatics Association
CAP: National Digital Health Capability Action Plan
