# Supplementary material for: Transforming the Future of Digital Health Education: Redesign of a Graduate Program Using Competency Mapping
Source: JMIR Med Educ. 2024 Oct 31;10:e54112. doi: 10.2196/54112 (PMC11542907; doi:10.2196/54112)
Supplement: Multimedia Appendix 1 [file mededu-v10-e54112-s001.docx]

| AMIA Domain / Competency | UOM ISYS 90069 (DTOH) | UOM INFO 90011 (ALHS) |
| --- | --- | --- |
|  |  |  |
| Domain I. Fundamentals: Fundamental knowledge and skills which provide clinical informaticians with a common vocabulary, basic knowledge across all Clinical Informatics domains, and understanding of the environment in which they function. |  |  |
| 1.1 Clinical Informatics |  |  |
| 1.1.1 The discipline of informatics | X |  |
| 1.1.2 Fundamental informatics concepts, models, and theories | X |  |
| 1.1.3 Core clinical informatics literature (e.g., foundational literature, principle journals, critical analysis of literature, use of evidence to inform practice) | X | X |
| 1.1.4 Descriptive and inferential statistics |  | X |
| 1.1.5 Health Information Technology (HIT) principles and science | X |  |
| 1.1.6 Computer programming fundamentals and computational thinking | X |  |
| 1.1.7 Basic systems and network architectures | X |  |
| 1.1.8 Basic database structure, data retrieval and analytics techniques and tools | X |  |
| 1.1.9 Development and use of interoperability/exchange standards (e.g., Fast Health Interoperability Resources [FHIR], Digital Imaging and Communications in Medicine [DICOM]) | X | X |
| 1.1.10 Development and use of transaction standards (e.g., American National Standards Institute X12) | X |  |
| 1.1.11 Development and use of messaging standards (e.g., Health Level Seven [HL7] v2) | X | X |
| 1.1.12 Development and use of ancillary data standards (e.g., imaging and Laboratory Information System [LIS]) | X | X |
| 1.1.13 Development and use of data model standards | X | X |
| 1.1.14 Vocabularies, terminologies, and nomenclatures (e.g., Logical Observation Identifiers Names and Codes [LOINC], Systematized Nomenclature of Medicine — Clinical Terms [SNOMED-CT], RxNorm, International Classification Of Diseases [ICD], Current Procedural Terminology [CPT]) | X | X |
| 1.1.15 Data taxonomies and ontologies | X | X |
| 1.1.16 Security, privacy, and confidentiality requirements and practices | X | X |
| 1.1.17 Legal and regulatory issues related to clinical data and information sharing | X | X |
| 1.1.18 Technical and non-technical approaches and barriers to interoperability | X | X |
| 1.1.19 Ethics and professionalism | X | X |
|  |  |  |
| 1.2 The Health System |  |  |
| 1.2.1 Primary domains of health, organizational structures, cultures, and processes (e.g., health care delivery, public health, personal health, population health, education of health professionals, clinical research) | X | X |
| 1.2.2 Determinants of individual and population health | X | X |
| 1.2.3 Forces shaping health care delivery and considerations regarding health care access | X | X |
| 1.2.4 Health economics and financing | X | X |
| 1.2.5 Policy and regulatory frameworks related to the healthcare systems | X | X |
| 1.2.6 The flow of data, information, and knowledge within the health system | X | X |
|  |  |  |
| Domain II. Improving Care Delivery and Outcomes: Knowledge and skills that enable a clinical informatician to develop, implement, evaluate, monitor, and maintain clinical decision support; analyze existing health processes and identify ways that health data and Health Information Systems (HIS) can enable improved outcomes; support innovation in the health system through informatics tools and processes. |  |  |
| 2.1 Decision science | X | X |
| 2.2 Clinical decision support standards and processes for development, implementation, evaluation, and maintenance | X | X |
| 2.3 Five Rights of clinical decision support (i.e., information, person, intervention formats, channel, and point/time in workflow) | X | X |
| 2.4 Legal, regulatory, and ethical issues regarding clinical decision support | X |  |
| 2.5 Methods of workflow analysis |  | X |
| 2.6 Principles of workflow re-engineering |  |  |
| 2.7 Quality improvement principles and practices | X | X |
| 2.8 User-centered design principles | X | X |
| 2.9 Usability testing |  | X |
| 2.10 Definitions of measures | X | X |
| 2.11 Measure development and evaluation processes and criteria | X | X |
| 2.12 Key performance indicators (KPIs) | X | X |
| 2.13 Claims analytics and benchmarks |  |  |
| 2.14 Predictive analytic techniques, indications, and limitations |  | X |
| 2.15 Clinical and financial benchmarking sources | X | X |
| 2.16 Quality standards and measures promulgated by quality organizations | X | X |
| 2.17 Facility accreditation quality and safety standards | X |  |
| 2.18 Clinical quality standards | X | X |
| 2.19 Reporting requirements | X | X |
| 2.20 Methods to measure and report organizational performance | X | X |
| 2.21 Adoption metrics |  | X |
| 2.22 Social determinants of health | X | X |
| 2.23 Use of patient-generated data | X | X |
| 2.24 Prediction models |  | X |
| 2.25 Risk stratification and adjustment |  | X |
| 2.26 Concepts and tools for care coordination | X | X |
| 2.27 Care delivery and payment models | X | X |
|  |  |  |
| Domain III. Enterprise Information Systems: Knowledge and skills that enable a clinical informatician to develop and deploy health information systems that are integrated with existing information technology systems across the continuum of care, including clinical, consumer, and public health domains. Develop, curate, and maintain institutional knowledge repositories while addressing security, privacy, and safety considerations. |  |  |
| 3.1 Health information technology landscape | X | X |
| 3.2 Institutional governance of clinical information systems | X | X |
| 3.3 Information system maintenance requirements |  | X |
| 3.4 Information needs analysis and information system selection | X | X |
| 3.5 Information system implementation procedures |  | X |
| 3.6 Information system evaluation techniques and methods | X | X |
| 3.7 Information system and integration testing techniques and methodologies |  | X |
| 3.8 Enterprise architecture (databases, storage, application, interface engine) | X |  |
| 3.9 Methods of communication between various software components | X |  |
| 3.10 Network communications infrastructure and protocols between information systems |  |  |
| 3.11 Types of settings | X |  |
| 3.12 Clinical system functional requirements |  | X |
| 3.13 Models and theories of human-computer (machine) interaction (HCI) | X |  |
| 3.14 HCI evaluation, usability engineering and testing, study design and methods | X | X |
| 3.15 HCI design standards and design principles | X | X |
| 3.16 Functionalities of clinical information systems | X | X |
| 3.17 Consumer-facing health informatics applications | X | X |
| 3.18 User types and roles, institutional policy and access control | X |  |
| 3.19 Clinical communication channels and best practices for use | X | X |
| 3.20 Security threat assessment methods and mitigation strategies | X |  |
| 3.21 Security standards and safeguards | X |  |
| 3.22 Clinical impact of scheduled and unscheduled system downtimes | X |  |
| 3.23 Information system failure modes and downtime mitigation strategies | X |  |
| 3.24 Approaches to knowledge repositories and their implementation and maintenance | X | X |
| 3.25 Data storage options and their implications | X | X |
| 3.26 Clinical registries | X | X |
| 3.27 Health information exchanges | X | X |
| 3.28 Patient matching strategies |  |  |
| 3.29 Master patient index |  |  |
| 3.30 Data reconciliation | X | X |
| 3.31 Regulated medical devices (e.g., pumps, telemetry monitors) that may be integrated into information systems | X | X |
| 3.32 Non-regulated medical devices (e.g., consumer devices) | X | X |
| 3.33 Telehealth workflows and resources (e.g., software, hardware, staff) | X | X |
|  |  |  |
| Domain IV. Data Governance and Data Analytics: Knowledge and skills that enable a clinical informatician to establish and maintain data governance structures, policies, and processes. Incorporate information from emerging data sources; acquire, manage, and analyze health-related data; ensure data quality and meaning across settings; and derive insights to optimize clinical and business decision making. |  |  |
| 4.1 Stewardship of data | X | X |
| 4.2 Regulations, organizations, and best practice related to data access and sharing agreements, data use, privacy, security, and portability | X | X |
| 4.3 Metadata and data dictionaries | X | X |
| 4.4 Data life cycle | X | X |
| 4.5 Transactional and reporting/research databases | X | X |
| 4.6 Techniques for storage of disparate data types | X | X |
| 4.7 Techniques to extract, transform, and load data |  | X |
| 4.8 Data associated with workflow processes and clinical context | X | X |
| 4.9 Data management and validation techniques |  | X |
| 4.10 Standards related to storage and retrieval from specialized and emerging data sources | X | X |
| 4.11 Types and uses of specialized and emerging data sources | X | X |
| 4.12 Issues related to integrating emerging data sources into business and clinical decision making | X | X |
| 4.13 Information architecture | X | X |
| 4.14 Query tools and techniques |  | X |
| 4.15 Flat files, relational and non-relational/NoSQL database structures, distributed file systems |  | X |
| 4.16 Definitions and appropriate use of descriptive, diagnostic, predictive, and prescriptive analytics |  | X |
| 4.17 Analytic tools and techniques |  | X |
| 4.18 Advanced modeling and algorithms |  | X |
| 4.19 Artificial intelligence | X | X |
| 4.20 Machine learning | X | X |
| 4.21 Data visualization |  | X |
| 4.22 Natural language processing | X | X |
| 4.23 Precision medicine (customized treatment plans based on patient-specific data) | X | X |
| 4.24 Knowledge management and archiving science |  |  |
| 4.25 Methods for knowledge persistence and sharing |  |  |
| 4.26 Methods and standards for data sharing across systems | X | X |
|  |  |  |
| Domain V. Leadership and Professionalism: Knowledge and skills that enable a clinical informatician to build support and create alignment for informatics best practices; lead health informatics initiatives and innovation through collaboration and stakeholder engagement across organizations and systems. |  |  |
| 5.1 Environmental scanning and assessment methods and techniques |  | X |
| 5.2 Consensus building, collaboration, and conflict management |  | X |
| 5.3 Business plan development for informatics projects and activities |  | X |
| 5.4 Basic revenue cycle |  |  |
| 5.5 Basic managerial/cost accounting principles and concepts |  | X |
| 5.6 Capital and operating budgeting |  | X |
| 5.7 Strategy formulation and evaluations |  | X |
| 5.8 Approaches to establishing Health Information Technology (HIT) mission and objectives |  | X |
| 5.9 Communication strategies, including one-on-one, presentation to groups, and asynchronous communications |  | X |
| 5.10 Effective communication programs to support and sustain systems implementation |  | X |
| 5.11 Writing effectively for various audiences and goals |  | X |
| 5.12 Negotiation strategies, methods, and techniques |  | X |
| 5.13 Conflict management strategies, methods, and techniques |  | X |
| 5.14 Change management principles, models, and methods |  | X |
| 5.15 Assessment of organizational culture and behavior change theories |  | X |
| 5.16 Theory and methods for promoting the adoption and effective use of clinical information systems |  | X |
| 5.17 Motivational strategies, methods, and techniques |  | X |
| 5.18 Basic principles and practices of project management |  | X |
| 5.19 Project management tools and techniques |  | X |
| 5.20 Leadership principles, models, and methods |  | X |
| 5.21 Intergenerational communication techniques |  |  |
| 5.22 Coaching, mentoring, championing and cheerleading methods |  | X |
| 5.23 Adult learning theories, methods, and techniques |  |  |
| 5.24 Teaching modalities for individuals and groups |  |  |
| 5.25 Methods to assess the effectiveness of training and competency development |  |  |
| 5.26 Principles, models, and methods for building and managing effective interdisciplinary teams |  | X |
| 5.27 Team productivity and effectiveness |  |  |
| 5.28 Group management processes |  | X |
