# Supplementary material for: Transforming the Future of Digital Health Education: Redesign of a Graduate Program Using Competency Mapping
Source: JMIR Med Educ. 2024 Oct 31;10:e54112. doi: 10.2196/54112 (PMC11542907; doi:10.2196/54112)
Supplement: Multimedia Appendix 2 [file mededu-v10-e54112-s002.docx]

| AIDH - Australian Health Informatics Competency Framework (2022) | UOM ISYS 90069 (DTOH) | UOM INFO 90011 (ALHS) |
| --- | --- | --- |
|  |  |  |
| A. Health sciences |  |  |
| A.1 Health concepts: Health informaticians outline the main concepts of physiology, anatomy, pathology, and the different levels of biological organisation.  Level: Understanding | X | X |
| A.2 Health determinants: Health informaticians explain the basic determinants of health.  Level: Understanding | X | X |
| A.3 Health language: Health informaticians interpret the meaning of health icons, terms, abbreviations, initialisms, and acronyms.  Level: Understanding | X | X |
| A.4 Clinical decision making: Health informaticians explain the processes of clinical decision making and diagnostic/therapeutic strategies.  Level: Understanding | X | X |
| A.5 Evidence informed practice: Health informaticians interpret health science evidence to inform decisions.  Level: Understanding | X | X |
| A.6 Care delivery: Health informaticians explain the nature, importance, and decision making process for continuity of care, shared care, and other models of healthcare delivery.  Level: Level: Understanding | X | X |
| A.7 Epidemiology concepts: Health informaticians summarise basic concepts of public health, health promotion, and epidemiology.  Level: Understanding | X | X |
| A.8 Health sector structures: Health informaticians describe how health systems are managed, funded, serviced, organised, and measured.  Level: Understanding | X | X |
| A.9 Health sector roles: Health informaticians differentiate the roles and responsibilities of health.  Level: Understanding | X | X |
|  |  |  |
| B. Information science |  |  |
| B.1 Information science concepts  Health informaticians articulate how concepts such as data, information, knowledge, and wisdom are used.  Level: Applying | X |  |
| B.2 Data and information analysis  Health informaticians employ the concepts of descriptive and inferential statistics and their utility to specific contexts.  Level: Applying |  | X |
| B.3 Data and information visualisation  Health informaticians identify effective visualisation methods for knowledge discovery and the representation of information.  Level: Understanding |  | X |
| B.4 Design and development  Health informaticians apply information science theories in the design and development of information systems.  Level: Applying | X | X |
| B.5 Implementation, adoption, and evaluation  Health informaticians apply information science theories in the implementation, adoption, and evaluation of information systems.  Level: Applying | X | X |
| B.6 Data governance principles  Health informaticians apply the principles of data governance to achieve quality data and information.  Level: Applying | X | X |
|  |  |  |
| C. Information technology |  |  |
| C.1 System design concepts  Health informaticians explain how knowledge of data structures, algorithms, and programming influence system design.  Level: Understanding | X | X |
| C.2 System lifecycle  Health informaticians apply appropriate, efficient and effective practices throughout the information system lifecycle.  Level: Applying | X | X |
| C.3 System security  Health informaticians select system security risks and mitigation strategies.  Level: Applying | X |  |
|  |  |  |
| D. Leadership and management |  |  |
| D.1 Leadership strategies: Health informaticians apply leadership strategies to digital health.  Level: Applying |  | X |
| D.2 Information and system governance: Health informaticians ascertain the appropriateness, ethics, effectiveness, and efficiency of information and information system governance.  Level: Applying | X | X |
| D.3 Business alignment: Health informaticians build alignment between information and information systems, and business/clinical goals and strategies.  Level: Applying |  | X |
| D.4 Stakeholder engagement: Health informaticians develop strategies for stakeholder engagement, applying relationship management principles.  Level: Applying |  | X |
| D.5 Program and project management: Health informaticians employ appropriate, effective, and efficient program and project management methodologies.  Level: Applying |  | X |
| D.6 Change management: Health informaticians employ appropriate, effective, and efficient change management methodologies.  Level: Applying |  | X |
| D.7 Risk management: Health informaticians employ appropriate, effective, and efficient risk management methodologies.  Level: Applying |  | X |
| D.8 Quality management: Health informaticians employ appropriate, effective, and efficient quality management methodologies.  Level: Applying |  | X |
| D.9 Value management and benefit realisation: Health informaticians select appropriate value management and benefit realisation strategies to support delivery of successful outcomes.  Level: Applying |  | X |
| D.10 Process reengineering: Health informaticians apply process reengineering to facilitate business and organisational transformation.  Level: Applying |  | X |
| D.11 Information culture: Health informaticians develop their organisation's information culture to contribute to a learning health system.  Level: Applying | X | X |
|  |  |  |
| E. Social and behavioural sciences |  |  |
| E.1 Sociotechnical concepts: Health informaticians illustrate informational, technological, and social systems for evidence informed decision making.  Level: Applying | X | X |
| E.2 Problem solving: Health informaticians apply problem solving methods for evidence informed decision making.  Level: Applying | X | X |
| E.3 Legislative and regulative requirements: Health informaticians interpret the legislative, regulatory, and policy obligations that are relevant in specific digital health contexts.  Level: Applying | X | X |
| E.4 Information privacy: Health informaticians determine best practice in the collection, use, disclosure, access, protection, and disposal of health information.  Level: Applying | X | X |
| E.5 User experience: Health informaticians determine best practice in human centred design, usability, human factors, and ergonomic sciences.  Level: Applying | X | X |
| E.6 Health literacy: Health informaticians determine best practice in integrating health literacy into information sources and systems.  Level: Applying | X | X |
|  |  |  |
| F. Core health informatics |  |  |
| F.1 Informatics history: Health informaticians relate the history of health informatics to the current and future health environment.  Level: Understanding | X |  |
| F.2 Informatics specialisation: Health informaticians differentiate areas of health informatics and specialisation.  Level: Analysing | X |  |
| F.3 Informatics theories: Health informaticians apply theoretical and conceptual frameworks to their practice.  Level: Applying | X | X |
| F.4 Health economic concepts: Health informaticians determine the economic impact of digital health.  Level: Applying | X | X |
| F.5 Stakeholder education: Health informaticians appraise the education and training needs of different stakeholders within the digital health context.  Level: Analysing | X | X |
| F.6 Information structure and design: Health informaticians appraise the structure and design of health information in different use contexts.  Level: Analysing | X | X |
| F.7 Data and information attributes: Health informaticians distinguish the key attributes of any type of data and information in relation to their intended or potential uses in health.  Level: Analysing | X | X |
| F.8 Emerging concepts: Health informaticians appraise new data sources and emerging technologies, and their relevance to health.  Level: Analysing | X | X |
| F.9 Data governance practices: Health informaticians distinguish approaches to health data governance, including sovereignty, in different contexts.  Level: Analysing | X | X |
| F.10 Indigenous Data Sovereignty: Health informaticians apply Indigenous Data Sovereignty principles to reduce the disparity in First Nations People health.  Level: Applying |  |  |
| F.11 Information system architectures: Health informaticians differentiate enterprise and information systems architecture components.  Level: Analysing | X | X |
